# The Young Parenting Inventory (YPI-R3), and the Baumrind, Maccoby and Martin Parenting Model: Finding Common Ground

**DOI:** 10.3390/children9020159

**Published:** 2022-01-26

**Authors:** John Philip Louis

**Affiliations:** Louis Counselling & Training Services, Pte. Ltd., 339159 Singapore, Singapore; johnphiliplouis@gmail.com

**Keywords:** Baumrind, Maccoby and Martin, parenting, second order, first order, factor analysis, confirmatory factor analysis, schema therapy, deviant, normal, abuse, neglect

## Abstract

The parenting typology of Baumrind, Maccoby and Martin is based on variations in warmth and control and consists of three negative parenting styles labelled authoritarian, neglectful, and permissive. This parenting typology is based on normal variations of parenting but did not include dimensions arising from deviant parenting (e.g., abuse and neglect). A parenting typology has emerged based on the schema therapy model through the development of the Young Parent Inventory (YPI-R3), which represents a fuller range of maladaptive parenting spanning the deviant to normal range of the parenting continuum. Using six international, community, nonclinical samples with separate ratings for mothers and fathers from the USA, *n* = 259, 281; South Africa, *n* = 318, 372; Nigeria, *n* = 328, 344; India, *n* = 277, 289; Singapore, *n =* 592, 628; and Malaysia, *n* = 222, 229, results showed that the best second order higher factor solution of the ten YPI-R3 subscales was a three factor solution that runs parallel to, and resembles, the three negative parenting styles of Baumrind, Macobby and Martin. This factor structure was also shown to be a consistent and cross-culturally acceptable model among the countries from which the samples were drawn. The resemblance and implications of both parenting models were discussed.

## 1. Introduction

In the 1960s, using qualitative analysis, Baumrind [1] uncovered three parenting styles, two of which were negative and one was positive. Later Maccoby and Martin [2] added a third negative construct called neglectful. The three negative parenting patterns were termed parenting styles and defined in terms of two dimensions (warmth and control) that create an emotional climate in which parents communicate their attitudes and practices about childrearing to their child [1,2]. These three negative parenting styles were based on variations in warmth and control and were labelled as authoritarian (low warmth–high control), permissive (high warmth–low control), and neglectful (low warmth–low control) [1,2]. Hundreds of studies [3] were conducted based on this parenting typology and while this proved to be extremely valuable over the decades it was based on normal or typical variations of parenting used to control and socialize children [4]. It did not include dimensions arising further towards the negative end of the parenting spectrum such as abuse and neglect. As a result there is a need to identify a fuller range of parenting patterns that includes both normal and deviant ones.

The Young Parent Inventory (YPI) [5] is arguably the most comprehensive scale (not psychometrically validated) measuring past parenting patterns. It was developed by Young et al. [5] for the treatment of the more difficult-to-treat patients, including those with personality disorders; populations hypothesized to have experienced traumatic and adverse childhood experiences. While normal or typical parenting patterns are described as predictable and expectable for a substantial number of families in a society, deviant parenting patterns consist of ongoing emotional or physical abuse, significant and sustained deprivation (e.g., of love, guidance, safety) or too much of a good thing (e.g., over protection). Schema therapy views such patterns as depriving a child of having his or her core emotional needs met adequately on a regular basis. Over time these failures are hypothesized to lead to the development of negative schemas which are linked to problematic behavioral dispositions that emerge during adolescence and adulthood. Negative schemas consist of memories, cognitions, beliefs, bodily sensations and neurobiological reactions regarding oneself and one’s relationship with others and, to date, 18 have been identified and empirically validated [6,7,8]. A total of 17 dysfunctional patterns of parenting were hypothesized in the YPI and items were developed for each. These items were derived based upon their hypothesized link to a negative schema [5,9]. The schema termed social isolation was not included since it was believed to originate from experiences with one’s peer group rather than early caregivers [5]. Since the YPI had not been validated, an initial pool of 204 items (72 items from the original YPI and 132 new items) was developed by Louis et al. [9]. After subjecting these to psychometric scrutiny, six subscales and 36 items emerged forming an improved version known as the YPI-R2. However, four negative parenting patterns were rejected during its development. New items were therefore developed for each of the rejected and weaker subscales and tested empirically. This process resulted in four additional subscales reaching a level of statistical significance commensurate with the original six and in a more comprehensive psychometrically validated version of the overall scale, the YPI-R3. This parenting scale consists of ten subscales and 41 items [10]. In comparing the ten negative parenting patterns of the YPI-R3 to the three broader ones from Baumrind, Maccoby and Martin’s parenting typology [1,2] its was hypothesized that the ten negative parenting patterns of the former would form three broader clusters paralleling the three constructs of the latter. This study sets out to test this hypothesis.

## 2. The Present Research

This study set out to test the hypothesis that the higher order factors of the ten YPI-R3 subscales cluster into three broader second order factors that resemble and run parallel to the three groups of Baumrind, Maccoby and Martin’s parenting typology [1,2], namely authoritarian, neglectful (or uninvolved), and permissive (or indulgent). Using the YPI-R3, a self-report questionnaire, samples were drawn from six nonclinical, international samples from Singapore, Malaysia, India, South Africa, Nigeria and the USA each with separate ratings for mothers and fathers. This resulted in a total of 12 samples. Using the Singapore sample correlations between the ten first-order factors of YPI-R3 would first be tested to see if they were substantial, and if so, then data would be deemed suitable for second order exploratory factor analysis (EFA) using the Singapore sample. Various second order models were tested. Thereafter, the most robust second order factor solution would be selected using confirmatory factor analysis (CFA) to test its model fit using the other five samples. Following this the model would also be tested to see if it was a cross-culturally acceptable one among the countries from which the samples were drawn [10,11]. A resemblance of the parenting typology obtained from the secondary structure of the YPI-R3 with the typology from Baumrind, Maccoby and Martin [1,2] would add a measure of qualitative and quantitative validity to both models and clarify the ways in which the more nuanced constructs of the schema therapy model relate to Baumrind, Maccoby and Martin’s negative parenting constructs [1,2].

## 3. Materials and Methods

### 3.1. Samples

Six international, non-clinical, English-speaking community samples (Singapore, Malaysia, the USA, South Africa, Nigeria, and India) with separate ratings for mothers and fathers (therefore 12 separate samples) were used in this cross-sectional study using self-report measures. The sample size (*n*), mean age, and standard deviation (SD) of the samples were as follows: the USA (*n* = 259, 281), 43.69 years, SD = 9.12; South Africa (*n* = 318, 372), 42.11 years, SD = 6.79; Nigeria (*n* = 328, 344), 45.7 years, SD = 7.19; India (*n* = 277, 289), 42.39 years, SD = 7.67; Singapore (*n* = 592, 628), 46.22 years, SD = 22.32, and Malaysia (*n* = 222, 229), 41.40, SD = 17.40. Except for the samples from India and Nigeria the number of women were greater than the number of men. This was likely due to the type of event and the incentives offered which attracted more females than males. The sample from Singapore was used for second order factor analysis using principal axis factoring. The other five international samples—USA, South Africa, Nigeria, India, and Malaysia—were used to assess model fit using CFA, as well as to test whether the model is a cross-cultural one.

Data collection was the same as reported in the Louis et al. [10] study where samples from Singapore and Malaysia were drawn from community during an in-person parenting workshop. For the remaining four samples data were obtained during the COVID-19 outbreak in 2020. During that period strict social distancing measures were implemented worldwide, therefore, the survey was conducted via online platforms. The ethics committee of the non-governmental organization (NGO) in each city provided consent to conduct this study among the volunteers and no one was excluded based on religion, color, or race. Notice of this survey was given about two months ahead of time through email dissemination. Those who agreed to participate provided informed consent. The notice contained information on the following: (i) the voluntary nature of the survey; (ii) the criteria for participating in the online survey being a minimum of 18 years of age; (iii) being currently married (in the online battery of tests there were questionnaires on marital issues for other research studies); (iv) being able to speak English; (v) confidentiality and the anonymity of the data; and (vi) the purpose of the research being for scientific publications. For the in-person workshops in Singapore and Malaysia, participants had a different battery of instruments and even though they had to be above 18 years of age and able to speak English they did not have to be married. As a result, this drew younger volunteers reflected in the SD being larger for these two samples (see Table 1).

These participants also invited their friends who, in turn, invited others and so a snowball effect was created increasing the number of participants. Incentives for the Singapore and Malaysia participants were a free book on parenting and a parenting workshop given after the survey. For the online participants the incentive was a two-hour free online parenting webinar during the COVID-19 outbreak. However, those who could not complete the online survey because they were not currently married, such as single parents, were not deprived of the incentives. Ethical standards were under the American Psychological Association and the British Psychological Society.

Online survey participants were requested to log in three weeks before the online parenting webinar to take the survey together although they were also given the choice to do so at their convenience. However, logging in collectively had the advantage that participants were able to raise any questions they may have and direct them to their respective group leaders. These leaders who were also present online were briefed beforehand by the author. For the online survey, all questions in the survey had to be answered and if there was no response to a question participants would be prompted to go back and respond to it. However, participants were allowed to reject the entire survey if they experienced distress in any way. Both surveys took about 45 mins to 1 hour on average for participants to complete and upon completion they were sent to the first author and an administrator. For the Singapore and Malaysia samples the surveys were conducted collectively in a quiet hall. All fields that can identify a participant, including the Internet Protocol addresses of participants, were removed and only the remaining data were used for analyses.

### 3.2. Measures

#### 3.2.1. YPI-R3

The most recent validated version of the initial theoretical YPI [5], known as the YPI-R3 [10], has ten subscales and 41 items that measure perceived past parenting experiences using a six-point Likert scale, ranging from 1 (*Completely untrue*) to 6 (*Describes him/her perfectly*). The ten subscales are labelled as: degradation and rejection, competitiveness and status seeking, over-control, emotional inhibition and deprivation, punitiveness and abuse, overprotection and overindulgence, undependability and irresponsibility, neglect and insufficient guidance, social exclusion, and intrusiveness and exploitation. Item examples are: “criticized me a lot” (degradation and rejection subscale), and “worried excessively that I would get hurt” (overprotection and overindulgence subscale). The initial YPI was developed based on the assumption that each negative schema originated from a corresponding unmet core emotional need resulting from a specific pattern of dysfunctional parenting [5]. A validated version of the YPI was developed, known as the YPI-R2 [9]. Four subscales representing more deviant and harmful parenting patterns were rejected in the process of developing and empirically validating the YPI-R2. New items were developed to strengthen these subscales. An empirical test of these new scales resulted in the development of an improved version known as the YPI-R3 [10]. As it was with the YPI and YPI-R2, scores on each subscale of the YPI-R3 are provided separately for ratings of mothers and fathers, or those whom the participants considered as having assumed a paternal or maternal role (grandparent, stepmother or father, or a much older sibling). This was performed to test the parenting outcome associated with the gender of the parent. The YPI-R3 [10] demonstrated solid psychometric properties where highly significant correlations (*p* < 0.01) were found between the ten subscales of the YPI-R3 and the 15 subscales/scales of Mini-International Personality Item Pool (IPIP), Gratitude Scale, Depression, Anxiety and Stress (DASS-21), Satisfaction with Life Scale (SWLS), Humor Styles Questionnaire (HSQ), and Eating Loss of Control Scale (ELOCS) with separate ratings of mothers and fathers [10]. The average statistically significant correlation values (|*r*|) in the fathers sample between the YPI-R3 scales and these measures were as follows: IPIP = 0.105; gratitude scale = 0.110; DASS-21 = 0.127; SWLS = 0.108; HSQ = 0.129; and ELOCS = 0.129. The values (|*r*|) for the mothers sample were: IPIP = 0.118; DASS-21 = 0.152; SWLS = 0.131; humor = 0.162; and ELOCS = 0.121 [10]. These effect sizes were small but significant [11]. However, effect sizes demonstrated by other established measures of past parenting patterns were of the same magnitude. For example, a parenting scale known as s-EMBU (Swedish acronym for “my memories of upbringing”) [12] with neuroticism measures, extraversion, and self-esteem were 0.20, 0.19, and 0.22, respectively. Similarly, Thimm [13] found significant correlations between s-EMBU and measures of personality disorder symptoms and depression with values of |*r*| = 0.26 and 0.22, respectively. Putnick et al. [14] also showed correlation values of the Parental Acceptance-Rejection Questionnaire with child adjustment measures that ranged from 0.06 to 0.14. Therefore, small [11] but statistically significant correlations are common between parenting scales with other measures of well-being, ill-being and emotional distress.

Hierarchical multiple regression showed that the YPI-R3 predicted statistically significant (*p* < 0.001) variance in 13 out of 15 dependent variables (IPIP, gratitude scale, DASS-21, SWLS, humor, and ELOCS) in the fathers sample, and 10 (eight with *p* < 0.001, two with *p* < 0.01) out of the 15 dependent variables in the mothers sample [10]. This was over and above the variance contributed by age and gender. The YPI-R3, therefore, has an impressive predictive capability for measures of personality traits (IPIP), emotional distress (DASS-21), psychopathology (ELOS), and other distal measures such as satisfaction with life, gratitude, and humor.

#### 3.2.2. Procedures and Statistical Analyses

IBM SPSS Statistics 23 (IBM Corp: Armonk, New York, USA) [15] and M*Plus* 8 software (Los Angeles, CA, USA) [16] were used to conduct all analyses. Before any analyses were conducted, missing data for the samples from Malaysia and Singapore were examined. Using Little’s Missing Completely at Random test [17] missing data analysis was carried out to see if missing patterns in both samples were random. However, Schafer [18] asserted that a missing rate of 5% or less is inconsequential. The values of skewness and kurtosis were calculated using the Singapore fathers and mothers sample. According to Hair et al. [19] values can be considered normal if skewness is between −2 to +2 and kurtosis is between −7 to +7. Notwithstanding this Tabachnick and Fidell [20] stated that if sample sizes were appreciably >200 (see Table 1) both CFA and EFA will be robust against violations of skewness and kurtosis.

Before an EFA was conducted parallel analysis (PA), an accurate and reliable method, was used to recommend the number of extracted factors [21]. EFA was then conducted using principal axis factoring (PAF) with promax rotation, an oblique rotation that allows factors to be correlated. Following these correlations between the first order factors of the ten YPI-R3 subscales were conducted to see if strength of correlations were at least medium in order to justify second order factor analysis. The threshold guidelines were as follows [15]: small (*r* = 0.10), medium (*r* = 0.30), and large effect sizes (*r* = 0.50).

Thereafter, CFA was used, and since CFA is considered a case of structural equation modeling certain assumptions must also be satisfied such as sample sizes being in excess of 200 according to Boomsma and Hoogland [22]. This was the case here for all 12 samples. CFA was performed using a weighted least-squares means and a variance adjusted estimation (WLSMV) algorithm to take into account the ordered-categorical nature of the response scales [23]. CFA also followed the guidelines in which a close fit is indicated by normed Chi-square, (*X^2^*/*df*) < 4; the root mean square error of approximation (RMSEA), where a reasonable fit by 0.06 < RMSEA < 0.08, a mediocre fit by 0.08 < RMSEA < 0.10, and an unacceptable fit by RMSEA > 0.10; comparative fit index (CFI), and one non-normed fit index known as the Tucker–Lewis (TLI) by values ≥0.95 for a good fit and ≥0.90 for an adequate fit [24,25]. Each model under examination needed to be further evaluated for acceptable fit based on prior findings. Floyd and Widaman [26] found that scales with high numbers of items and factors generally lead to a poorer fit. This was evident from three studies; Bach et al. [7], Baranoff et al. [27], and Kriston et al. [8], where the YSQ-S3 (90 items) were subjected to CFA, in which the CFI values obtained were below the 0.9 threshold with values of 0.84, 0.87, and 0.85, respectively (the values of *X^2^*/*df* and RMSEA in these studies were above the recommended minimum threshold). Thus more relaxed values for indices may be considered an acceptable fit for such scales; for example, a value for CFI and TLI that is slightly less than 0.90 can be viewed as a moderate fit in studies with a large number of items. Equally, for scales with a small number of items, it would be appropriate to adopt more stringent fit criteria [26]. Given the number of factors and items, we determined a priori to accept the lower bound of fit values as well fitting in the context since there were a large number of items (41 items) in the YPI-R3 scale.

For second order analysis recommendations made by Bryne [28] would be used for its justification. They were that (a) the higher order model represents a well-fitting model; (b) the discrepancies in fit indices between first and second order models are minimal; (c) that correlations among the first order factors are substantial; and (d) there is a theoretical justification to consider a higher order construct. Following this, multigroup CFA (MGCFA) would be conducted to see if the model is consistent cross-culturally by demonstrating invariance. To achieve this, guidelines by Milfont and Fischer [29] were used: the same measurement patterns for the latent constructs, the same psychological meanings for the latent constructs, the same levels of the latent constructs. Therefore, the following measurements of invariance using MGCFA were used for the five samples (USA, South Africa, Nigeria, India, and Malaysia): (1) configural invariance (same factor structure across groups); (2) metric invariance (same factor loadings across groups); (3) scalar invariance (same item intercepts across groups); (4) error invariance (same error variance across groups); (5) factor variance/invariance (same factor variance across groups); (6) factor covariance (same factor covariance across groups), and (7) factor mean invariance (same factor mean across groups). If invariance is obtained at all levels, it would show that the model is a consistent and cross-culturally acceptable one among the countries from which the samples were drawn [29].

## 4. Results

The percentages of missing data from the samples from Malaysia and Singapore were very low; 0.07% and 0.06% respectively. In accordance with Schafer’s [18] recommendation that a missing rate of 5% or less would be inconsequential the mean values were used to impute missing data values. Due to nature of the survey and the way it was organized there were no missing data from samples obtained online, namely USA, South Africa, Nigeria and India. The values of skewness and kurtosis were within range of a normal distribution as recommended by Hair et al. [19] (see Supplementary Tables S1 and S2). The correlations values were large (>0.5) [14] for some of first-order factors (see Supplementary Tables S3 and S4). On the basis of these large effect sizes second-order analyses was deemed suitable for the data [28]. Before proceeding with second order analysis PA was conducted which recommended four second order factors to be extracted from the mothers as well as the fathers samples from Singapore. However, a four-factor solution did not indicate a robust pattern as there were three cross-loaded items for the mothers sample and two for the fathers samples (see Table 2).

**Figure 1:**
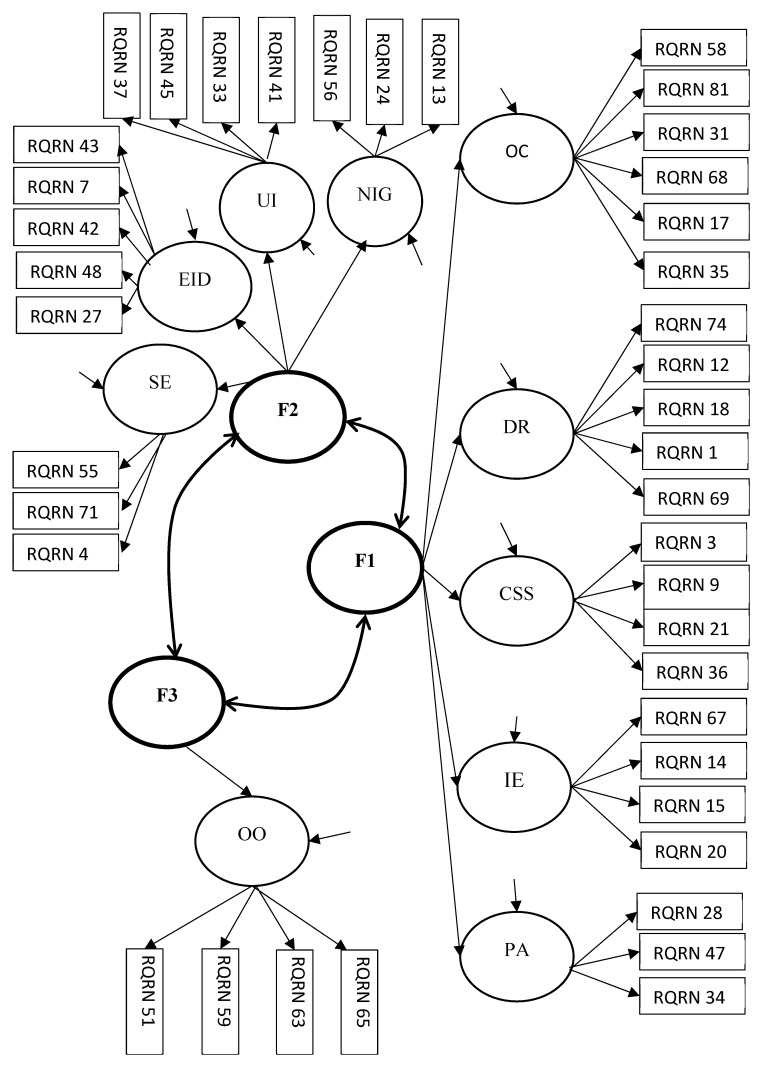
The Hypothesized Three Factor, Second Order Confirmatory Factor Analysis Model of the YPI-R3. OC = over-control; EID = emotional inhibition and deprivation; UI = undependability and irresponsibility; OO = overprotection and overindulgence; NIG = neglect and insufficient guidance; CSS = competitiveness and status seeking; IE = intrusiveness and exploitation; DR = degradation and rejection; SE = social exclusion; PA = punitiveness and abuse. F1 = authoritarian and/or abusive (second order of YPI-R3); F2 = neglectful and/or undependable (second order of YPI-R3); F3 = overprotective and overindulgent (second order of YPI-R3). RQRN = Item identification letters uniquely used in this study.

The four-factor solution also consisted of items whose loadings were less than the 0.4 cut off value. When a three-factor solution was forced it yielded a clear solution with no cross-loaded items for both the mothers and fathers samples (see Table 3 and Figure 1). Further, the loadings of all items were > 0.4. The three factors were labelled as (a) authoritarian and/or abusive since it consisted of the following first order factors of the YPI-R3—over-control, degradation and rejection, competitiveness and status seeking, punitiveness and abuse, and intrusiveness and exploitation; (b) neglectful and/or undependable since it consisted of the following first order factors of the YPI-R3—neglect and insufficient guidance, emotional inhibition and deprivation, undependability and irresponsibility, and social exclusion; and (c) overprotective and overindulgent, which emerged by itself. As hypothesized, they resembled and ran in parallel with the three broader groups of Baumrind, Maccoby, and Martin’s’ parenting typology respectively, namely authoritarian, neglectful, and permissive [1,2].

A two-factor solution was also tested but this did not produce satisfactory results because the overprotection and overindulgence subscale did not appear (see Table 4).

In examining the four, three and two factor models, the three-factor model was the most robust and therefore the best solution (Figure 1) with no cross-loaded items and inclusion of all the first order items in the factor solution. Confirmatory factor Analyses was the used to check the model fit for the three-factor solution of the second order structure of the YPI-R3 (see Table 5) using five other international samples. The results are shown in Table 5.

The results of the CFA showed that the fit was reasonable based on RMSEA and the normed Chi-square, (*X^2^*/*df*) were all <4, from 2.10 to 2.84), and adequate fit based on CFI and TLI values although some of these values were slightly below the cutoff point of the lower bound 0.9 value. As mentioned Floyd and Widaman [26] found that scales with high numbers of items and factors generally lead to a poorer fit. This was evident from three studies; Bach et al. [7], Baranoff et al. [27], and Kriston et al. [8], where the YSQ-S3 (90 items) were subjected to CFA and the CFI values obtained were below the 0.9 threshold with values of 0.84, 0.87, and 0.85 respectively. Finally, results from MGCFA showed that invariance was obtained at all seven levels which showed that the model is a consistent and cross-culturally acceptable one among the countries from which the samples were drawn (see Table 6 and Table 7) [6,12].

Finally, the CFA fit indices of the ten first order factors and the three second order factors were compared with one another (see Table 8). Results showed that the fit indices for the first order factors of the YPI-R3 was slightly better overall than the second order factors showing that data were represented better by the ten first order factors [28].

## 5. Discussion

The parenting typology of Baumrind [1], and Maccoby and Martin [2], based on variations of warmth and control, consists of three negative parenting styles—authoritarian (low warmth–high control), neglectful (low warmth–low control), and permissive (high warmth–low control). These have been proven to be extremely valuable as hundreds of studies have been conducted that show associations between these parenting styles and negative outcomes in children [3]. These parenting styles, however, were based on normal variations in parenting, not those from deviant parenting patterns such as abuse and neglect [4]. Since consideration was not given to children raised in home environment where more deviant parenting patterns are normative, new measures that can identify a fuller range of parenting patterns must be developed so that parents, clinicians and educators can derive the necessary insights as to the type of unhealthy parenting parents from both a normal well as a more harmful or deviant home environment.

The YPI-R3 filled this gap with ten maladaptive parenting constructs and consists of both normal (typical) and deviant (atypical) parenting patterns. The ten negative parenting patterns are identified as: degradation and rejection, competitiveness and status seeking, over-control, emotional inhibition and deprivation, punitiveness and abuse, overprotection and overindulgence, undependability and irresponsibility, neglect and insufficient guidance, social exclusion, and intrusiveness and exploitation [10]. Based on a study of the YPI-R3 by Louis et al. [10], six out of the ten subscales of the YPI-R3, namely degradation and rejection, overprotection and overindulgence, undependability and irresponsibility, neglect and insufficient guidance, social exclusion, and intrusiveness and exploitation yielded the largest effect sizes with negative schemas. Thus, these are likely to represent the more deviant parenting patterns since the strength of negative schemas are associated with the level of abuse, neglect and toxicity in the home environment as reported and supported by the schema therapy model [5,6,9,10]. The other subscales are more likely to be found in normal or typical homes, though these can also inflict harm if such practices are taken to extremes.

This study had set to see if there is a link between the three broad constructs of the parenting typology of Baumrind, Maccoby and Martin with a second order factor solution of the ten YPI-R3 subscales. Samples were diverse and drawn from six international, nonclinical populations—USA, Nigeria, South Africa, India, Malaysia, and Singapore. These represented Eastern, African and Western samples. Singapore and USA represented samples from developed countries while samples from Nigeria, South Africa, India and Malaysia represented the developing world. Using EFA, four, three, and two factor solutions were produced but the most robust one was a three-factor solution that did not contain any cross loaded items, and it included all ten of the YPI-R3 subscales. These three broad factors were labelled authoritarian and/or abusive (consisting of the first order YPI-R3 subscales of over-control, degradation and rejection, competitiveness and status seeking, punitiveness and abusive and intrusiveness and exploitation); neglectful and/or undependable (consisting of the first order YPI-R3 subscales of neglect and insufficient guidance, emotional inhibition and deprivation, undependability and irresponsibility, and social exclusion); and overprotective and overindulgent. The YPI-R3 subscale overindulgence and overprotection emerged by itself and did not cluster with any of the other YPI-R3 subscales. Inspection of these three broader categories from the YPI-R3 paralleled the three parenting styles from the Baumrind, Maccoby and Martin parenting typology [1,2]—authoritarian (Baumrind, Maccoby and Martin’s typology) paralleling authoritarian and/or abusive (YPI-R3 second order); neglectful or uninvolved (Baumrind, and Maccoby and Martin’s typology) paralleling neglectful and/or undependable (YPI-R3 second order); and permissive or indulgent (Baumrind, Maccoby and Martin’s typology) paralleling overprotective and overindulgent (YPI-R3 second order). This showed convergence of both parenting models developed from two very different vantage points; Baumrind’s model was developed qualitatively from direct observation of parents and the YPI-R3 was developed quantitatively from items that were based on adults’ recollections of their interactions with parents or caretakers in the course of schema therapy. Such convergence showed that the more nuanced and numerous deviant parenting patterns can be grouped into broader categories that have the advantage of being simpler for parents and educators to remember and track, being easier to measure, and if a scale was developed based upon these three broad constructs it would be shorter and take less time and energy for participants to fill it out. However, within a clinical context, the first order factors are more advantageous in that they serve as a clearer and more precise guide for interventions. In addition the results of the CFA show that the first order factors of the YPI-R3 fit the data better. Identifying more nuanced parenting patterns will help understand the specific nature of these parenting patterns. Take for example the maladaptive construct of “authoritarian and/or abusive”, one of three broader (second order factor) categories of the YPI-R3 scale. A child may experience this in a variety of ways—not being allowed to make age appropriate choices (over-control subscale of the YPI-R3), being shamed in front of others constantly (degradation and rejection subscale of the YPI-R3), being put down when not reaching a certain standard academically (competitiveness and status seeking subscale of the YPI-R3), punished physically (punitiveness and abuse subscale of the YPI-R3), or being sexually abused (intrusiveness and exploitation subscale of the YPI-R3). By delineating the “authoritarian and/or abusive” more clearly parents and clinicians will better understand what is going wrong and why. Having established both a first and second order structure will help parents and clinicians have the advantages of the brevity of the broader patterns and the precision offered by the first order structure.

The convergence arrived at between these two models strengthens both and is a counterpoint to the frequent failures in replication that has become more common in the field of psychology. For example, one report showed that only 36% of the studies in psychology replicated their original results on samples taken from new cultures [30]. In addition, also of note is that the factor solution that consisted of the three second order factors from the ten YPI-R3 scale was replicated in the fathers as well as in the mothers sample. This underscored that the role of fathers should not be minimized, and that fathers, similar to mothers, contribute to the perception of past parenting patterns, be it normal or deviant, as recalled by adults of their childhood who participated in this study.

## 6. Limitations

The first was that incentives to attend a workshop on parenting may have been the reason significantly more women than men signed up to take part in the study. This sample bias could limit the generalizability of the findings. Second, the methods used to collect data were from self-report surveys and participants may have been biased when reporting their own experiences.

## 7. Future Studies

Since the YPI-R3 was developed to measure both normal and deviant parenting patterns, and since the latter has been documented to be associated with the development of personality disorders future studies should be conducted using clinical samples. The second order factor solution of the YPI-R3 should also be tested in other countries and cultures using younger as well as with older participants. A shorter scale can also now be developed tapping into the most robust items of the ten YPI-R3. These items can be grouped into the three broader categories which, if empirically validated, can provide a shorter scale measuring normal and deviant negative parenting patterns.

As important as this new measure of dysfunctional parenting are measures of exceptional parenting. To meet this need an instrument known as the positive parenting schema inventory (PPSI) [31,32] in a manner parallel to the YPI-R3, provides a broader and more nuanced picture of all that goes well in parenting than any of the other current models or measures. It offers an empirically validated framework of seven positive parenting patterns. The YPI-R3 is an important guide with respect to what not to do. Knowing what to do does not always flow obviously from knowing what not to do. The PPSI addresses the need for a clearer guide to healthy parenting. An area for further study will be a similar effort to expand upon and replicate the PPSI as was performed in moving from the YPI-R2 to the YPI-R3 and a similar exploration of the second order structure. In addition, knowing what exceptional parents do and what kinds of difference this makes in developmental outcomes will be an important area of study. In this way the subject of healthy parenting can move in the direction of becoming more fully integrated into the field of positive psychology. In many respects, it could be seen as an exploration of the roots of positive psychology.

## Figures and Tables

**Table 1:** * Demographic Characteristics of Samples.

Characteristics	Categories	USA	South Africa	Nigeria	India	Singapore	Malaysia
Gender	Men	147	159	209	169	260	83
	Women	249	231	155	137	371	149
	Total	396	390	364	306	631	232
Age	Mean	43.69	42.11	45.7	42.39	46.22	41.40
	SD	9.12	6.79	7.19	7.67	22.34	17.40
Missing	>10%	0	0	0	0	3	3
Race	Chinese	N.A.	N.A.	N.A.	N.A.	508	205
	Indonesian	N.A.	N.A.	N.A.	N.A.	5	5
	Indian	N.A.	7	N.A.	N.A.	15	3
	Filipino	N.A.	N.A.	N.A.	N.A.	91	9
	Caucasian / White	104	65	N.A.	N.A.	2	2
	Black	52	135	N.A.	N.A.	N.A.	N.A.
	Latino	121	N.A.	N.A.	N.A.	N.A.	N.A.
	Asian	99	N.A.	N.A.	N.A.	N.A.	N.A.
	Colored	N.A.	17	N.A.	N.A.	N.A.	N.A.
	Yoruba	N.A.	N.A.	191	N.A.	N.A.	N.A.
	Ibo	N.A.	N.A.	72	N.A.	N.A.	N.A.
	Hausa	N.A.	N.A.	5	N.A.	N.A.	N.A.
	North India	N.A.	N.A.	N.A.	31	N.A.	N.A.
	East India	N.A.	N.A.	N.A.	44	N.A.	N.A.
	South India	N.A.	N.A.	N.A.	138	N.A.	N.A.
	West India	N.A.	N.A.	N.A.	45	N.A.	N.A.
	Others	20	7	96	48	9	8
	Did not specify	0	159	0	0	1	0
	Missing	0	0	0	0	3	3
	Sample Size	396	390	364	306	628	229
	** Final Fathers Sample Size, *n*	259	318	328	277	592	222
	** Final Mothers Sample Size, *n*	281	372	344	289	628	229

* This table was taken from the study by Louis et al. [10] ** Final fathers sample removed participants from the sample who did not grow up with a father; ** final mothers sample removed participants from the sample who did not grow up with a mother.

**Table 2:** Four-Factor Solution for YPI-R3 using Singapore Mothers and Fathers Sample.

Mothers						Fathers		
YPI-R3 First Order Factors	Factors					Factors		
	1	2	3	4	1	2	3	4
OC	0.834				0.876			
DR	0.782				0.806			
CSS	0.782				0.806			
PA	0.761			0.340	0.787			
IE	0.452		0.371		0.631		0.481	
NIG		0.853				0.813		
EID		0.498	0.400			0.800		
UI		0.477					0.913	
SE			0.767			0.460	0.630	
OO				0.541				0.979

Extraction method: principal axis factoring; rotation method: promax with Kaiser normalization; OC = over-control; EID = emotional inhibition and deprivation; UI = undependability and irresponsibility; OO = overprotection and overindulgence; NIG = neglect and insufficient guidance; CSS = competitiveness and status seeking; IE = intrusiveness and exploitation; DR = degradation and rejection; SE = social exclusion; PA = punitiveness and abuse.

**Table 3:** Three-Factor Solution for YPI-R3 using the Singapore Mothers and Fathers Sample.

	Mothers Sample			Fathers Sample			
YPI-R3 First Order Factors	Factor				Factor		
	1	2	3		1	2	3
OC	0.859			OC	0.902		
DR	0.814			DR	0.839		
CSS	0.786			CSS	0.826		
PA	0.769			PA	0.816		
IE	0.465			IE	0.610		
NIG		0.882		NIG		0.927	
EID		0.494		EID		0.663	
UI		0.480		UI		0.608	
SE		0.433		SE		0.581	
OO			0.608	OO			0.979

OC = over-control; EID = emotional inhibition and deprivation; UI = undependability and irresponsibility; OO = overprotection and overindulgence; NIG = neglect and insufficient guidance; CSS = competitiveness and status seeking; IE = intrusiveness and exploitation; DR = degradation and rejection; SE = social exclusion; PA = punitiveness and abuse.

**Table 4:** Two-Factor Solution for YPI-R3 using Singapore Mothers and Fathers Sample.

Mothers Sample			Fathers Sample		
YPI-R3 First Order Factors	Factor			Factor	
	1	2		1	2
OC	0.869		OC	0.901	
DR	0.797		CSS	0.868	
CSS	0.763		DR	0.821	
PA	0.723		PUN	0.784	
IE	0.439		IE	0.587	
NIG	−0.345	0.885	NIG		0.919
EID		0.536	EID		0.638
UI		0.463	UI		0.630
SE		0.419	SE		0.569
OO			OO		

OC = over-control; EID = emotional inhibition and deprivation; UI = undependability and irresponsibility; OO = overprotection and overindulgence; NIG = neglect and insufficient guidance; CSS = competitiveness and status seeking; IE = intrusiveness and exploitation; DR = degradation and rejection; SE = social exclusion; PA = punitiveness and abuse.

**Table 5:** CFA results for Three-Factor Second Order Solution for YPI-R3.

Model	Number of Parameters	*χ^2^*	*df*	*p*	*χ^2^/df*	CFI	TLI	RMSEA (CI)
**Fathers**								
Malaysia	258	1643.812	767	<0.001	2.14	0.88	0.87	0.072 (0.067–0.077)
USA	258	1807.136	767	<0.001	2.36	0.90	0.90	0.072 (0.068–0.077)
South Africa	258	2066.145	767	<0.001	2.69	0.93	0.92	0.073 (0.069–0.077)
Nigeria	258	1750.401	767	<0.001	2.28	0.94	0.94	0.063 (0.059–0.066)
India	258	1880.033	767	<0.001	2.45	0.89	0.88	0.072 (0.068–0.077)
**Mothers**								
Malaysia	258	1616.696	767	<0.001	2.11	0.88	0.87	0.070 (0.065–0.074)
USA	258	1650.019	767	<0.001	2.15	0.91	0.90	0.064 (0.060–0.068)
South Africa	258	2001.574	767	<0.001	2.61	0.94	0.93	0.066 (0.062–0.069)
Nigeria	258	2181.266	767	<0.001	2.84	0.91	0.90	0.073 (0.070–0.077)
India	258	1820.338	767	<0.001	2.37	0.90	0.89	0.069 (0.065–0.073)

**Table 6:** MG CFA Results Using Mothers Samples from USA, South Africa, Nigeria, India, and Malaysia.

Model	Number of Parameters	*χ^2^*	*df*	*p*	*χ^2^/df*	CFI	TLI	RMSEA		
									Comparison	Decision
Configural invariance	1250	9509.20	3875	<0.001	2.45	0.908	0.903	0.069 (0.068–0.071)	-	Accept
Metric invariance	1102	9679.93	4023	<0.001	2.41	0.908	0.906	0.068 (0.066–0.070)	Configural vs. metric	Accept
		(308.22)	(148)	<0.001		(<0.001)	(−0.003)	(−0.001)		
Scalar invariance	494	10055.77	4631	<0.001	2.17	0.912	0.922	0.062 (0.061–0.064)	Metric vs. scalar	Accept
		(1224.78)	(608)	(<0.001)		(−0.004)	(−0.016)	(−0.006)		
Residual variance invariance	290	9966.466	4835	<0.001	2.06	0.916	0.929	0.059 (0.058–0.061)	Scalar vs. residual	Accept
		(606.35)	(204)	(<0.001)		(−0.004)	(−0.007)	(−0.003)		
Factor variance invariance	282	9062.66	4843	<0.001	1.87	0.931	0.942	0.054 (0.052–0.055)	Residual vs. factor variance	Accept
		(30.01)	(8)	(<0.001)		(−0.015)	(−0.013)	(−0.005)		
Factor covariance invariance	270	8609.47	4855	<0.001	1.77	0.939	0.948	0.051 (0.049–0.052)	Factor variance vs. factor covariance	Accept
		(95.78)	(12)	(<0.001)		(−0.008)	(−0.006)	(−0.003)		
Factor mean invariance	264	9034.14	4861	<0.001	1.86	0.932	0.943	0.053 (0.052–0.055)	Factor covariance vs. factor mean	Accept
		(105.49)	(6)	(<0.001)		(0.007)	(0.005)	(0.002)		
Acceptance criteria for indices (differences)						>0.9	>0.9	<0.06		
						(<0.01)	(<0.01)	(<0.015)		

**Table 7:** MG CFA Results Using Fathers Samples from USA, South Africa, Nigeria, India, and Malaysia.

Model	Number of Parameters	*χ^2^*	*df*	*p*	*χ^2^/df*	CFI	TLI	RMSEA		
									Comparison	Decision
Configural invariance	1250	9388.05	3875	<0.001	2.42	0.914	0.909	0.071 (0.069–0.073)		Accept
Metric invariance	1102	9692.57	4023	<0.001	2.41	0.911	0.910	0.071 (0.069–0.073)		
		(515.75)	(148)	(<0.001)		(0.003)	(−0.001)	(<0.001)	Configural vs. metric	Accept
Scalar invariance	494	10349.93	4631	<0.001	2.23	0.910	0.921	0.066 (0.065–0.068)		
		(1497.71)	(608)	(<0.001)		(0.001)	(−0.011)	(−0.005)	Metric vs. scalar	Accept
Residual variance invariance	290	10183.63	4835	<0.001	2.11	0.916	0.929	0.063 (0.061–0.064)		
		(695.97)	(204)	(<0.001)		(−0.006)	(−0.008)	(−0.003)	Scalar vs. residual	Accept
Factor variance invariance	282	9696.67	4843	<0.001	2.00	0.924	0.936	0.060 (0.058–0.061)		
		(53.56)	(8)	(<0.001)		(−0.008)	(−0.007)	(−0.003)	Residual vs. factor variance	Accept
Factor covariance invariance	270	9359.75	4855	<0.001	1.93	0.929	0.940	0.057 (0.056–0.059)		
		(109.77)	(12)	(<0.001)		(−0.005)	(−0.004)	(−0.003)	Factor variance vs. factor covariance	Accept
Factor mean invariance	264	9740.37	4861	<0.001	2.00	0.924	0.936	0.060 (0.058–0.062)		
		(93.32)	(6)	(<0.001)		(0.005)	(0.004)	(0.003)	Factor covariance vs. factor mean	Accept

**Table 8:** Fit Indices for the Three-Second Order Factor Solution and the Ten First Order Solution of the YPI-R3.

	The Second Order Solution of YPI-R3				The First Order Solution of YPI-R3			
	*χ^2^/df*	CFI	TLI	RMSEA	*χ^2^/df*	CFI	TLI	RMSEA
**Fathers**								
Malaysia	2.14	0.875	0.866	0.072 (0.067–0.077)	3.48	0.915	0.905	0.065 (0.062–0.068)
USA	2.36	0.902	0.895	0.072 (0.068–0.077)	1.87	0.909	0.898	0.063 (0.058–0.068)
South Africa	2.69	0.927	0.922	0.073 (0.069–0.077)	2.13	0.922	0.913	0.066 (0.062–0.071)
Nigeria	2.28	0.942	0.939	0.063 (0.059–0.066)	2.40	0.942	0.936	0.066 (0.062–0.070)
India	2.45	0.89	0.880	0.072 (0.068–0.077)	2.23	0.910	0.900	0.067(0.062–0.071)
**Mothers**								
Malaysia	2.11	0.880	0.872	0.070 (0.065– 0.074)	1.77	0.920	0.911	0.058 (0.053–0.063)
USA	2.15	0.906	0.899	0.064 (0.060– 0.068)	2.02	0.920	0.911	0.060 (0.056–0.065)
South Africa	2.61	0.936	0.932	0.066 (0.062–0.069)	2.36	0.949	0.942	0.060 (0.057–0.064)
Nigeria	2.84	0.907	0.901	0.073 (0.070–0.077)	2.51	0.928	0.919	0.066 (0.062–0.070)
India	2.37	0.900	0.890	0.069 (0.065–0.073)	2.13	0.921	0.912	0.062 (0.058–0.067)

## Data Availability

The data presented in this study are available on request from the corresponding author. The data are not publicly available due in accordance with consent provided by participants on the use of confidential data.

## Supplementary Materials

The following are available online at https://www.mdpi.com/article/10.3390/children9020159/s1, Table S1: Descriptive Statistics of YPI-R3 Subscales Using Singapore Mothers Sample, Table S2: Descriptive Statistics of YPI-R3 Subscales Using Singapore Fathers Sample, Table S3: Pearson’s Correlation Between the First Order YPI-R3 Constructs for Singapore Mothers Sample (*n* = 628), Table S4: Pearson’s Correlation Between the First Order YPI-R3 Constructs for Singapore Fathers Sample (*n* = 592).
