# Supplementary material for: The Young Parenting Inventory (YPI-R3), and the Baumrind, Maccoby and Martin Parenting Model: Finding Common Ground
Source: Children (Basel). 2022 Jan 26;9(2):159. doi: 10.3390/children9020159 (PMC8870261; doi:10.3390/children9020159)
Supplement: Supplementary file 1 [file children-09-00159-s001.zip › children-1461388-supplementary.pdf]

Supplementary Material

Table S1. Descriptive Statistics of YPI-R3 Subscales Using Singapore Mothers Sample.

	**OC**	**EID**	**UI**	**OO**	**NIG**	**CSS**	**IE**	**DR**	**SE**	**PA**
Size (*n*)	628	628	628	628	628	628	628	628	628	628
Mean	2.794	3.306	1.877	2.290	3.055	2.950	1.466	2.355	2.441	2.496
Kurtosis	-.463	-.638	2.028	.422	-.829	-.633	5.479	.107	.035	.170
Skewness	.575	.176	1.353	.893	.214	.389	2.149	.925	.793	.978

Table S2. Descriptive Statistics of YPI-R3 Subscales Using Singapore Fathers Sample

	**OC**	**EID**	**UI**	**OO**	**NIG**	**CSS**	**IE**	**DR**	**SE**	**PA**
Size (*n*)	592	592	592	592	592	592	592	592	592	592
Mean	2.605	3.506	2.082	2.009	3.287	2.751	1.483	2.294	2.624	2.333
Kurtosis	.154	-.808	1.065	.918	-.862	-.226	5.052	.308	-.449	.493
Skewness	.883	.001	1.251	1.030	.163	.598	2.076	1.052	.636	1.115

OC=Over-Control
EID=Emotional Inhibition and Deprivation
UI=Undependability and Irresponsibility
OO=Overprotection and Overindulgence
NIG=Neglect and Insufficient Guidance
CSS=Competitiveness and Status Seeking
IE=Intrusiveness and Exploitation
DR=Degradation and Rejection
SE=Social Exclusion
PA=Punitiveness and Abuse

Table S3

Pearson's Correlation Between the First Order YPI-R3 Constructs for Singapore Mothers Sample (*n*=628).

	**OC**	**EID**	**UI**	**OO**	**NIG**	**CSS**	**IE**	**DR**	**SE**	**PA**
OC	1	.371**	.313**	.081*	.010	.594**	.452**	.705**	.350**	.703**
EID	.371**	1	.257**	-.048	.415**	.152**	.331**	.444**	.482**	.481**
UI	.313**	.257**	1	.105**	.388**	.138**	.427**	.298**	.306**	.350**
OO	.081*	-.048	.105**	1	.073	.197**	.025	.018	.109**	-.092*
NIG	.010	.415**	.388**	.073	1	-.181**	.195**	.126**	.289**	.147**
CSS	.594**	.152**	.138**	.197**	-.181**	1	.288**	.533**	.248**	.424**
IE	.452**	.331**	.427**	.025	.195**	.288**	1	.508**	.364**	.525**
DR	.705**	.444**	.298**	.018	.126**	.533**	.508**	1	.408**	.748**
SE	.350**	.482**	.306**	.109**	.289**	.248**	.364**	.408**	1	.384**
PA	.703**	.481**	.350**	-.092*	.147**	.424**	.525**	.748**	.384**	1

Table S4

Pearson's Correlation Between the First Order YPI-R3 Constructs for Singapore Fathers Sample (*n*=592)

	**OC**	**EID**	**UI**	**OO**	**NIG**	**CSS**	**IE**	**DR**	**SE**	**PA**
OC	1	.334**	.237**	.072	-.014	.617**	.481**	.715**	.295**	.726**
EID	.334**	1	.235**	-.076	.420**	.168**	.234**	.412**	.473**	.442**
UI	.237**	.235**	1	.017	.462**	.112**	.365**	.278**	.249**	.276**
OO	.072	-.076	.017	1	-.021	.189**	.002	.031	.065	-.044
NIG	-.014	.420**	.462**	-.021	1	-.187**	.151**	.147**	.335**	.133**
CSS	.617**	.168**	.112**	.189**	-.187**	1	.335**	.555**	.179**	.460**

IE	.481**	.234**	.365**	.002	.151**	.335**	1	.531**	.240**	.539**
DR	.715**	.412**	.278**	.031	.147**	.555**	.531**	1	.343**	.742**
SE	.295**	.473**	.249**	.065	.335**	.179**	.240**	.343**	1	.344**
PA	.726**	.442**	.276**	-.044	.133**	.460**	.539**	.742**	.344**	1

OC=Over-Control
EID=Emotional Inhibition and Deprivation
UI=Undependability and Irresponsibility
OO=Overprotection and Overindulgence
NIG=Neglect and Insufficient Guidance

CSS=Competitiveness and Status Seeking
IE=Intrusiveness and Exploitation
DR=Degradation and Rejection
SE=Social Exclusion
PA=Punitiveness and Abuse
